# Supplementary material for: Colibactin produced by a honeybee symbiont defends against pathogens and shapes the gut community
Source: bioRxiv. 2025 Dec 27:2025.12.27.695564. Preprint. [Version 1] doi: 10.64898/2025.12.27.695564 (PMC12990990; doi:10.64898/2025.12.27.695564)
Supplement: Supplement 2 [file NIHPP2025.12.27.695564v1-supplement-2.pdf]

bioRxiv preprint doi: https://doi.org/10.64898/2025.12.27.695564; this version posted December 27, 2025. The copyright holder for this preprint (which was not certified by peer review) is the author/funder, who has granted bioRxiv a license to display the preprint in perpetuity. It is made available under aCC-BY 4.0 International license.

Supplementary Information for

Colibactin produced by a honeybee symbiont defends against pathogens and shapes the gut community

Yulin Song[1]*, J. Elijah Powell[1], Joel W. H. Wong[2], Yunxi Liu[2], Miguel A. Aguilar Ramos[2], Tyler De Jong[1], Patrick J. Lariviere[1,3], Jayaditya Maganti[1], Emily P. Balskus[2,4], Nancy A. Moran[1]*

* Corresponding author. Email: nancy.moran@austin.utexas.edu (N. A. Moran), yulinsong450@gmail.com (Y. Song).

The PDF file includes:

- Supplementary Text
- Supplementary Figs. S1 to S18
- Supplementary Tables S1 to S4
- Supplementary Data S1
- Supplementary References

bioRxiv preprint doi: https://doi.org/10.64898/2025.12.27.695564; this version posted December 27, 2025. The copyright holder for this preprint (which was not certified by peer review) is the author/funder, who has granted bioRxiv a license to display the preprint in perpetuity. It is made available under aCC-BY 4.0 International license.

Supplementary Text

The colibactin locus is ubiquitous in *F. perrara* genomes

So far there are only two sequenced isolates of *F. perrara*, strain PEB0191 (DSM 104328) from hives in Connecticut USA (*1*) and strain ESL0167 from Lausanne Switzerland (*2*). Both have the colibactin locus (Supplementary Fig. S1). There are also 9 *F. perrara* metagenome-assembled genomes (MAGs), which may not be complete, and 6 of these have the locus (Supplementary Fig. S1). The locus is absent in genomes of all other honeybee gut bacteria.

F. perrara stimulates hindgut ROS to a similar level as other symbionts

Measurement of ROS in hindguts of gnotobiotic honeybees demonstrated that colonization by *F. perrara* increased ROS to a similar level as did three other bee gut symbionts, *S. alvi*, *G. apicola*, and *G. apis* ($p > 0.38$ between the symbionts), which were all higher than MD ($p < 0.01$) (Supplementary Fig. S4).

Elevated ROS generates the first line of pathogen defense and may explain the protective effect shortly after challenge (at day 1) from both WT and *ΔclbB* (Fig. 1D). However, the longer-term protection is mostly attributed to colibactin, as it is conferred by WT but not by *ΔclbB.* Bee gut symbionts modulate ROS production, which functions in determining host-symbiont specificity (*3*) and may also contribute to the observed protection against *S. marcescens* in previous studies *(4, 5)*.

In mammalian systems, colibactin production appears to have complex effects on host immune responses. In murine models, *pks*$^{+}$ *E. coli* did not alter intestinal inflammation under homeostatic conditions (*6*) but caused colibactin-dependent chronic colitis after a chemical disruption of mucosal integrity (*7*). On the other hand, the probiotic *E. coli* Nissle 1917 reduced intestinal inflammation of chemically induced colitis in a colibactin-dependent way (*8*). These studies suggest that effects of colibactin may vary depending on the producing strain and on gut homeostasis. Similarly, in *F. perrara,* colibactin may have multiple effects on host immune responses. Further study on the correlation between colibactin production by *F. perrara* and host immune responses will help to elucidate their potential interplay and combined effects on host health.

Other attempts at investigating effects of *F. perrara* and colibactin on bee gut symbionts

We constructed pSL1-E2C-B2P$_{recA}$eGFP, in which the promoter region of the *S. alvi recA* gene homolog was used for DNA damage reporting. However, it was not effective as MMC treatment failed to trigger a GFP signal (Supplementary Fig. S11). Three prophage-like islands were found in the genome of *S. alvi* wkB2 (Supplementary Fig. S12), but they were not induced by MMC or by *F. perrara* (Supplementary Fig. S13).

F. perrara colonization has limited impact on the normal bee gut community

We inoculated a defined community of 8 core and 1 non-core (*Bartonella*) strains (Table S1) to gnotobiotic bees inoculated with *F. perrara* WT, *ΔclbB* or no *F. perrara* 5 days in advance (Supplementary Fig. S18A). Community structures in the hindguts of individual bees after another 5 days were estimated as relative and absolute abundances, using 16S rRNA gene amplicons classified into genera (Supplementary Fig. S18B and C).

bioRxiv preprint doi: https://doi.org/10.64898/2025.12.27.695564; this version posted December 27, 2025. The copyright holder for this preprint (which was not certified by peer review) is the author/funder, who has granted bioRxiv a license to display the preprint in perpetuity. It is made available under aCC-BY 4.0 International license.

F. perrara ΔclbB colonized less than WT, which is consistent with the prior CFU results. In general, absolute and relative abundances of taxa were similar across the three different *F. perrara* treatments. The exception was for *Gilliamella* which was negatively correlated with *Frischella,* whether WT or *ΔclbB* (p = 0.97), in linear regression analysis (Supplementary Fig. S18D). We reanalyzed community composition data of natural hive bees from two published studies (*9, 10*), and again found negative correlations between abundances of these two taxa (Supplementary Fig. S18E and F).

Supplementary Figures

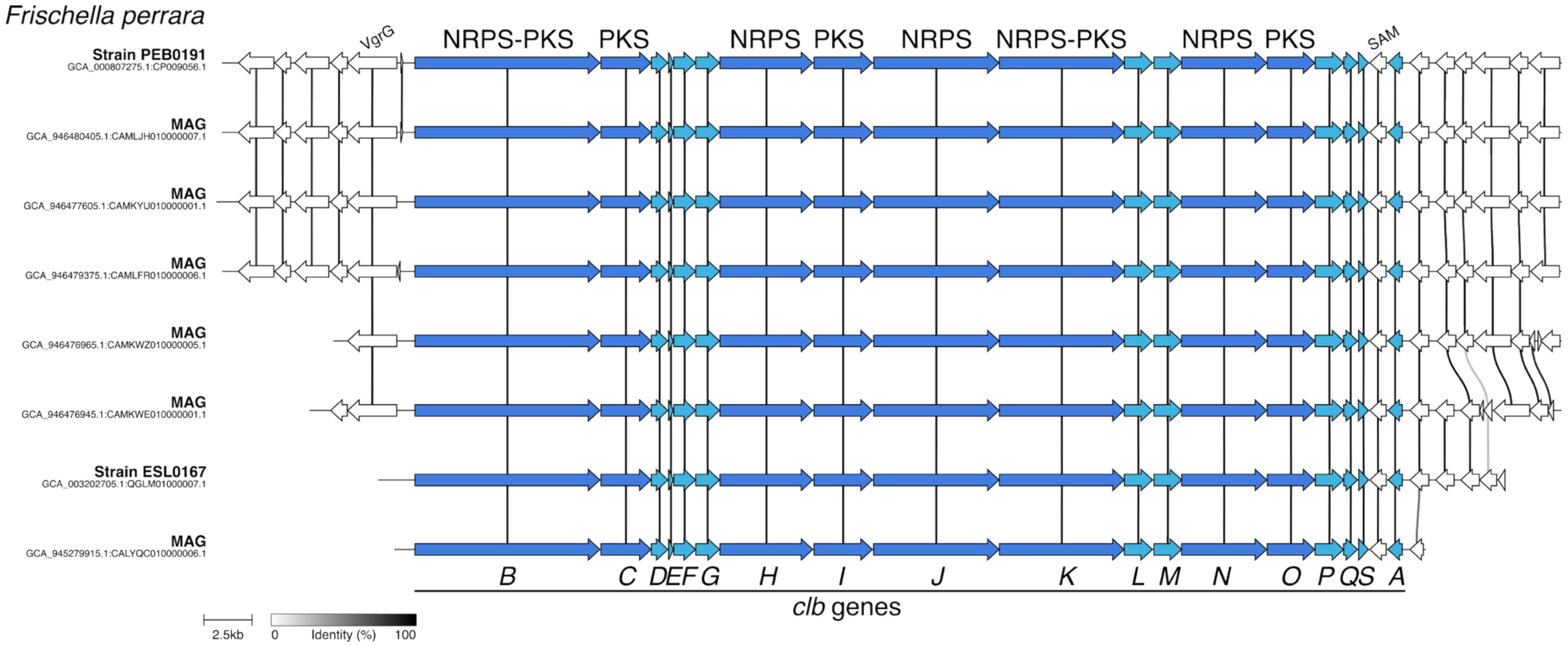

Fig. S1. *Frischella perrara* genomes possess the highly conserved colibactin locus, with the exception of 3 metagenome-assembled genomes (MAGs: GCA_946699615.1, GCA_946477445.1, and GCA_025291255.1). Homologous genes are linked with lines, and line color depth indicates the percent amino acid identity as indicated by the key. Colibactin genes are in blue. Gene names (*clbA–S*) are given below. *F. perrara* (strain PEB0191) genes/proteins are detailed in the GenBank annotation (accession: NZ_CP009056.1). Abbreviations for neighboring genes: *VgrG*, type VI secretion system tip protein; SAM, radical *S*-adenosylmethionine protein; hypothetical proteins (white).

bioRxiv preprint doi: https://doi.org/10.64898/2025.12.27.695564; this version posted December 27, 2025. The copyright holder for this preprint (which was not certified by peer review) is the author/funder, who has granted bioRxiv a license to display the preprint in perpetuity. It is made available under aCC-BY 4.0 International license.

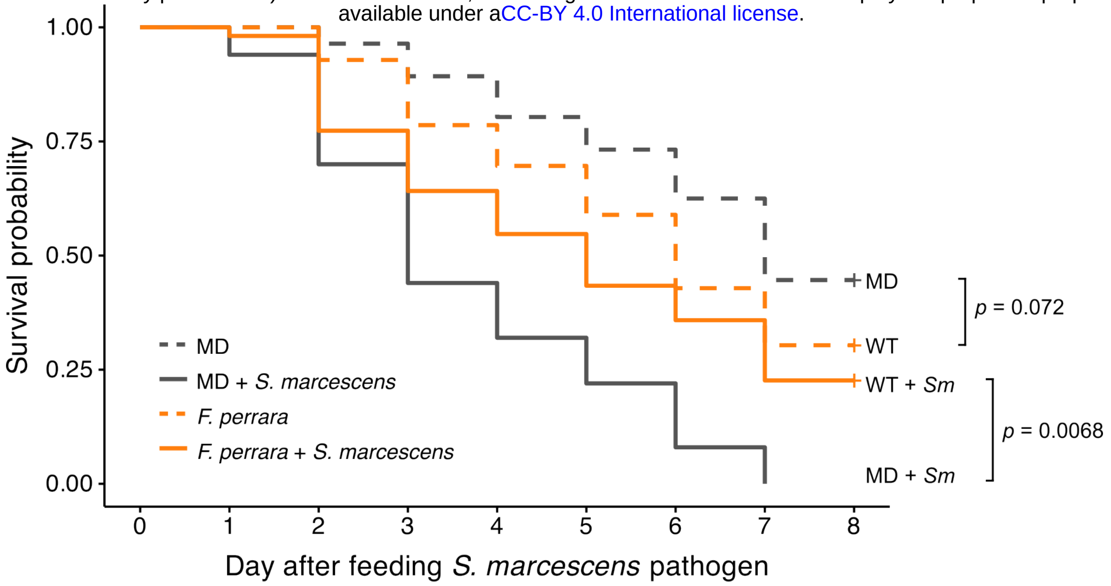

Fig. S2. Survival of honeybees after pathogen challenge. Newly emerged bees initially received inoculation of either *Frischella perrara* symbiont or PBS control (microbiota-deprived, MD). The inoculation was conducted in 50 mL falcon tubes with breath holes. 150 µL cell suspension at OD_{600} of 1 in PBS were mixed with 150 µL sucrose water (*v/v*, 50:50) and added to tubes containing about 20 bees. Tubes were gently shaked to ensure the contact of bees with the mixture and kept statically for 5 min. The bees were subsequently moved to cup cages. After 5 days (day 0 in the plot), the bees were fed with the pathogen *Serratia marcescens* or PBS. Total n = 215 bees. p-values were calculated using a pairwise log-rank test with Benjamini–Hochberg correction.

bioRxiv preprint doi: https://doi.org/10.64898/2025.12.27.695564; this version posted December 27, 2025. The copyright holder for this preprint (which was not certified by peer review) is the author/funder, who has granted bioRxiv a license to display the preprint in perpetuity. It is made available under aCC-BY 4.0 International license.

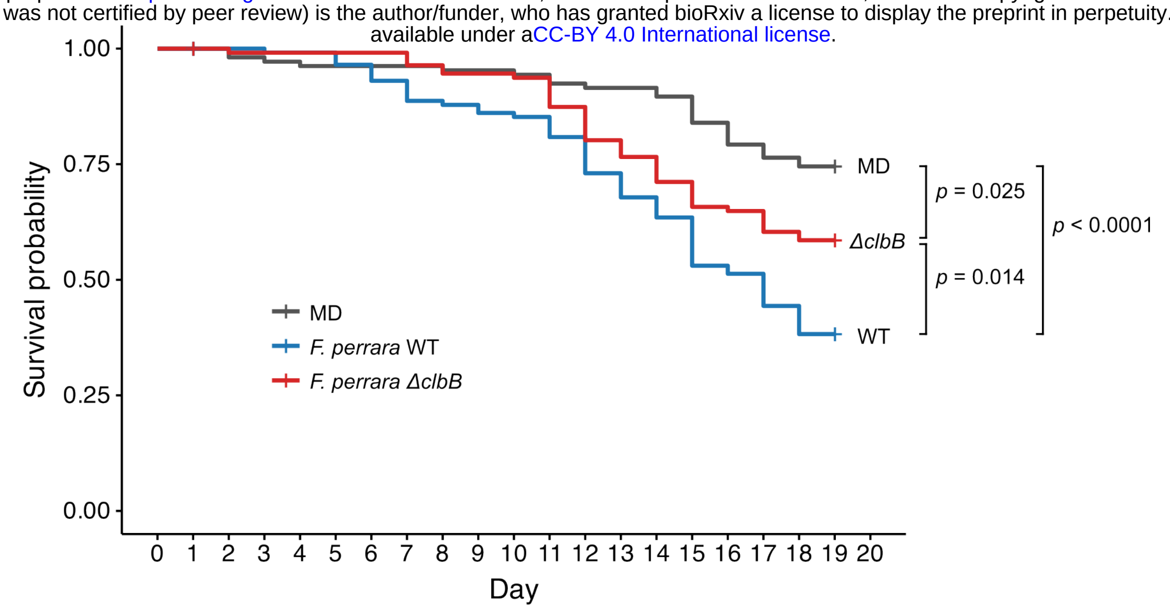

Fig. S3. Survival of honeybees inoculated with *F. perrara* WT, *F. perrara ΔclbB* or not inoculated (MD). The Kaplan-Meier survival curves combined two independent experiments with bees sourced from different hives (~20 bees per cup cage, 3 cages per group in each experiment). ANOVA test (χ^2 = 27.75, *df* = 2, $p < 0.0001$, n = 345) showed statistical significance among the treatment groups. Post-hoc pairwise comparisons were conducted using estimated marginal means.

bioRxiv preprint doi: https://doi.org/10.64898/2025.12.27.695564; this version posted December 27, 2025. The copyright holder for this preprint (which was not certified by peer review) is the author/funder, who has granted bioRxiv a license to display the preprint in perpetuity. It is made available under aCC-BY 4.0 International license.

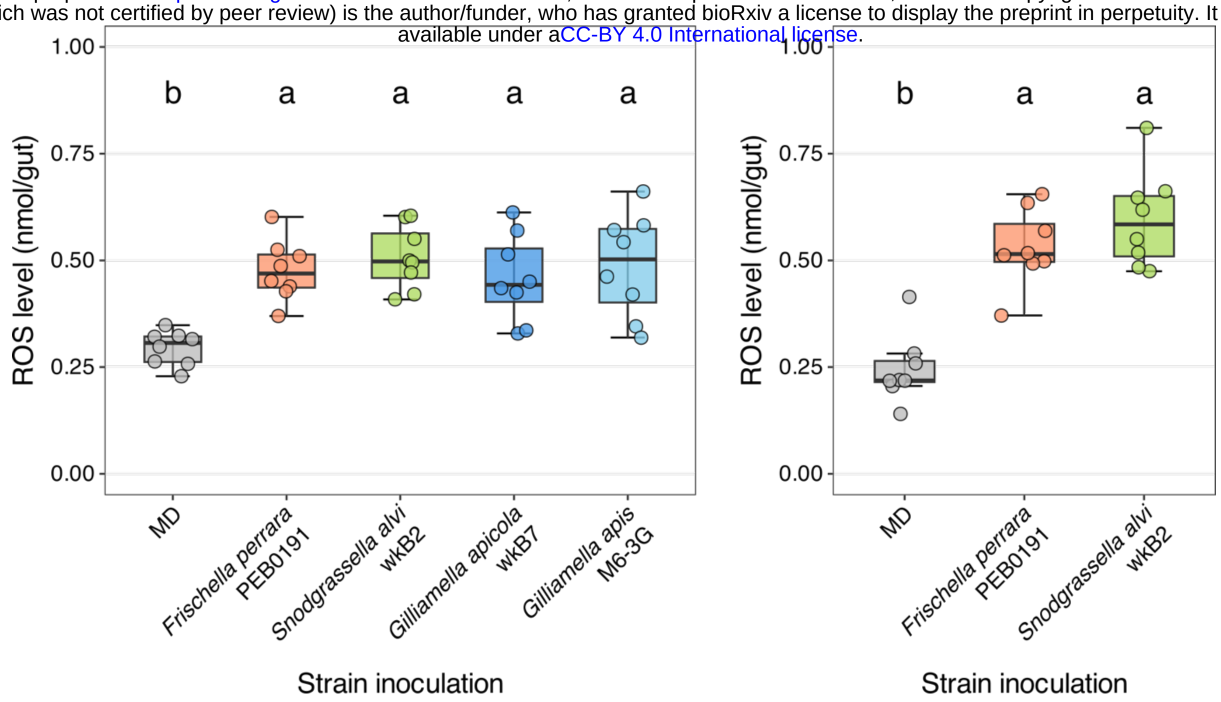

Fig. S4. *F. perrara*, and other bee gut symbionts, increase ROS level in the hindgut of gnotobiotic honeybees. Results were from two independent experiments. ANOVA test ($F(4, 35) = 7.903$, $p < 0.001$, $n = 40$; $F(2, 21) = 30.855$, $p < 0.0001$, $n = 24$) showed statistical significance among groups in both experiments. Post-hoc pairwise comparisons were conducted using a Tukey's pairwise comparisons test. Groups with different letters are significantly different ($\alpha = 0.05$).

bioRxiv preprint doi: https://doi.org/10.64898/2025.12.27.695564; this version posted December 27, 2025. The copyright holder for this preprint (which was not certified by peer review) is the author/funder, who has granted bioRxiv a license to display the preprint in perpetuity. It is made available under aCC-BY 4.0 International license.

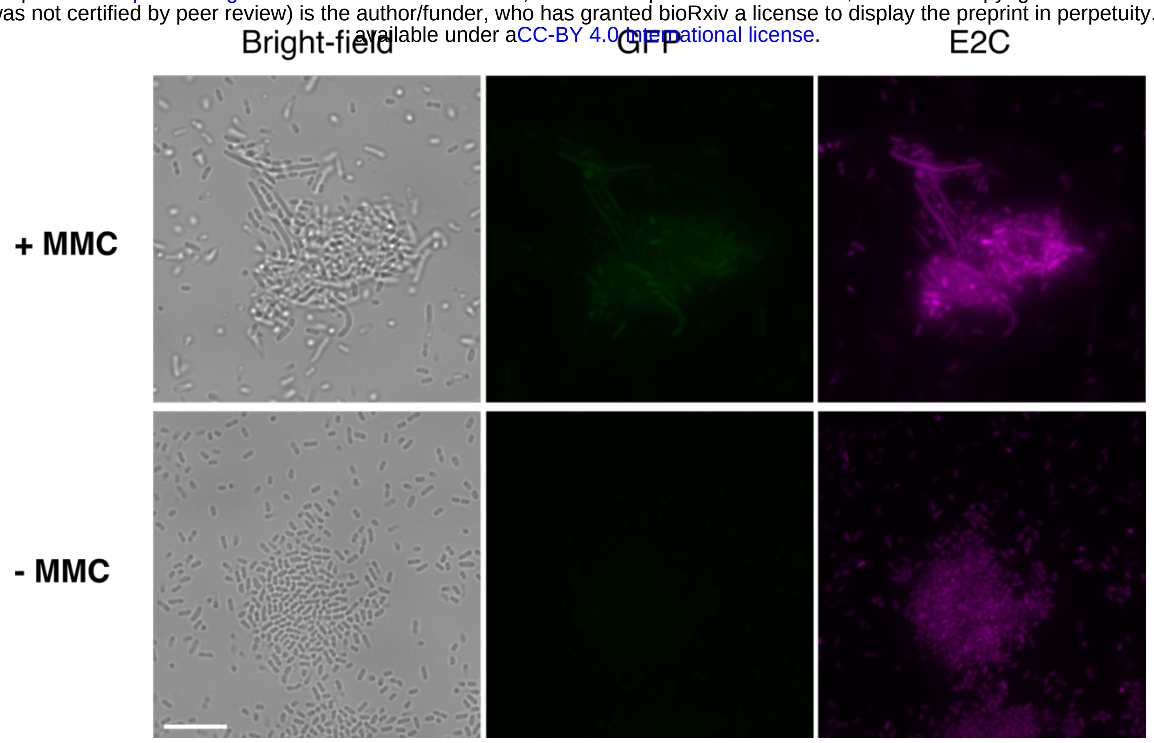

Fig. S5. Mitomycin C (MMC, 0.05 µg/mL) treatment for 5 hours induced GFP in *S. marcescence* carrying pSL1-E2C-N10P$_{recA}$eGFP. The elongated morphotype in the treated group is probably caused by arrested cell cycle. Scale bar = 10 µm.

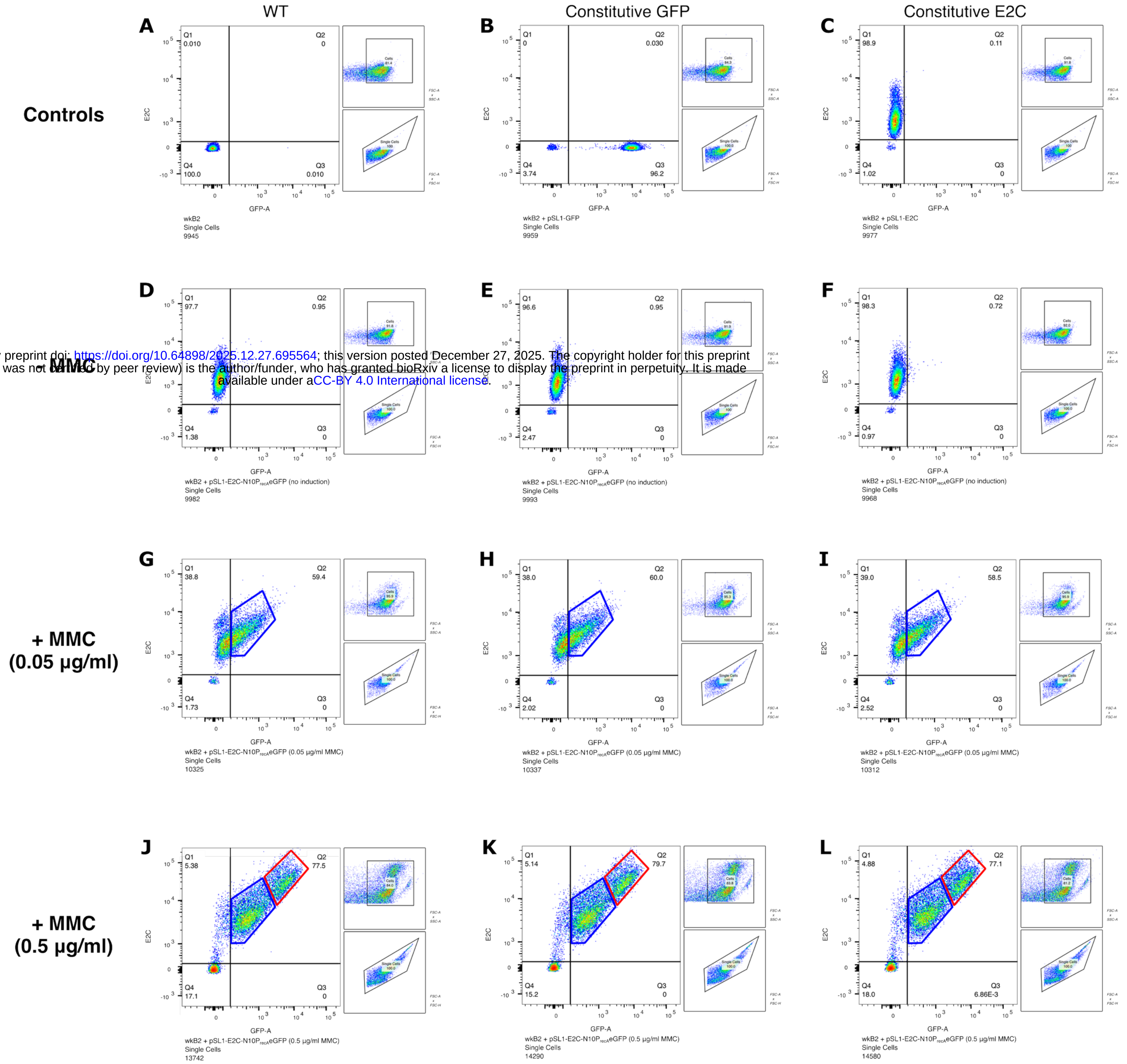

bioRxiv preprint doi: https://doi.org/10.64898/2025.12.27.695564; this version posted December 27, 2025. The copyright holder for this preprint (which was not certified by peer review) is the author/funder, who has granted bioRxiv a license to display the preprint in perpetuity. It is made available under aCC-BY 4.0 International license.

Fig. S6. Fluorescence flow cytometry analysis of *S. marcescens* carrying pSL1-E2C-N10P$_{recA}$eGFP, with or without MMC treatment. The left chart of each panel shows the intensity of GFP (x axis) and E2C (y axis), and right charts show the ancestry gating steps. The 4 quarters (Q1-4) were divided based on fluorescent and non-fluorescent controls (A-C). Q2 is positive in both GFP and E2C. *S. marcescens* plus pSL1-E2C-N10P$_{recA}$eGFP was treated with no MMC (D-F), low MMC (G-I), or high MMC (J-L), three replicates each. Both low and high concentrations of MMC triggered a population with increased GFP intensity (blue gated). High concentration triggered another population with both GFP and E2C intensities increased (red gated) and also more cells that lost both fluorescence in Q4.

bioRxiv preprint doi: https://doi.org/10.64898/2025.12.27.695564; this version posted December 27, 2025. The copyright holder for this preprint (which was not certified by peer review) is the author/funder, who has granted bioRxiv a license to display the preprint in perpetuity. It is made available under aCC-BY 4.0 International license.

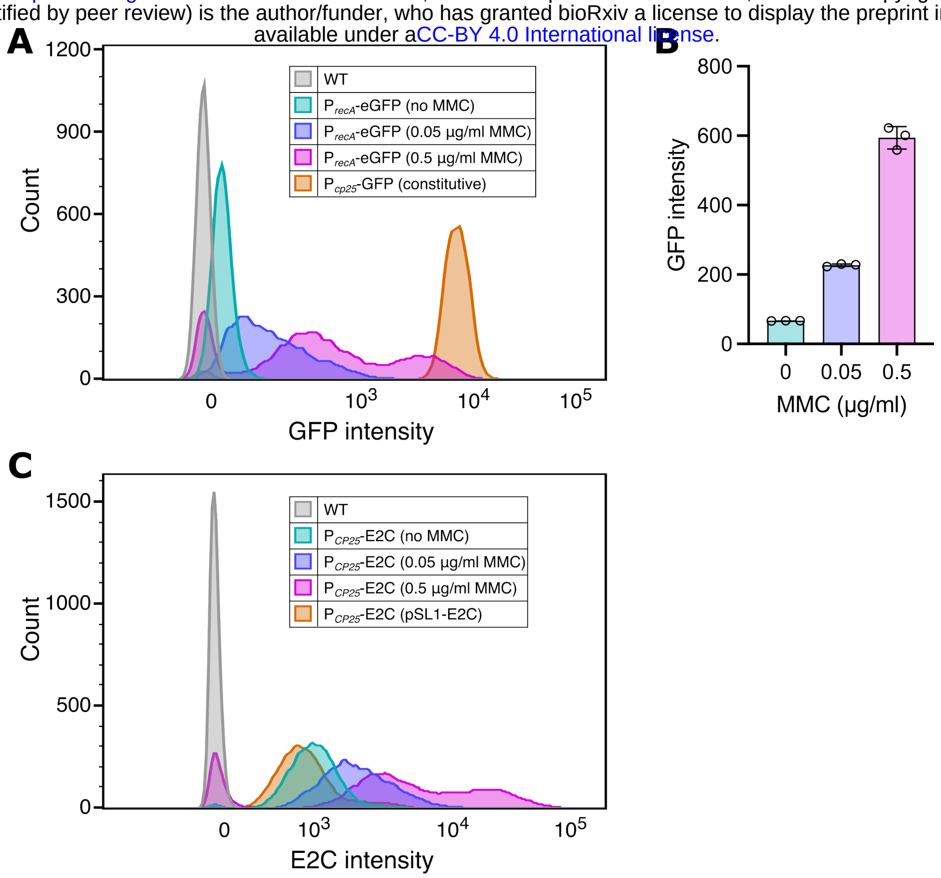

Fig. S7. Fluorescence intensities of *S. marcescens* carrying pSL1-E2C-N10P$_{recA}$eGFP, with or without MMC treatment. (A) GFP intensity under different MMC concentrations. WT and WT + pSL1-GFP (constitutive GFP) were used as controls. (B) Median GFP intensity (± SD) increased with MMC concentration. (C) E2C intensity under different MMC concentrations. WT and WT + pSL1-E2C were used as controls. All samples (except WT) constitutively express E2C.

bioRxiv preprint doi: https://doi.org/10.64898/2025.12.27.695564; this version posted December 27, 2025. The copyright holder for this preprint (which was not certified by peer review) is the author/funder, who has granted bioRxiv a license to display the preprint in perpetuity. It is made available under aCC-BY 4.0 International license.

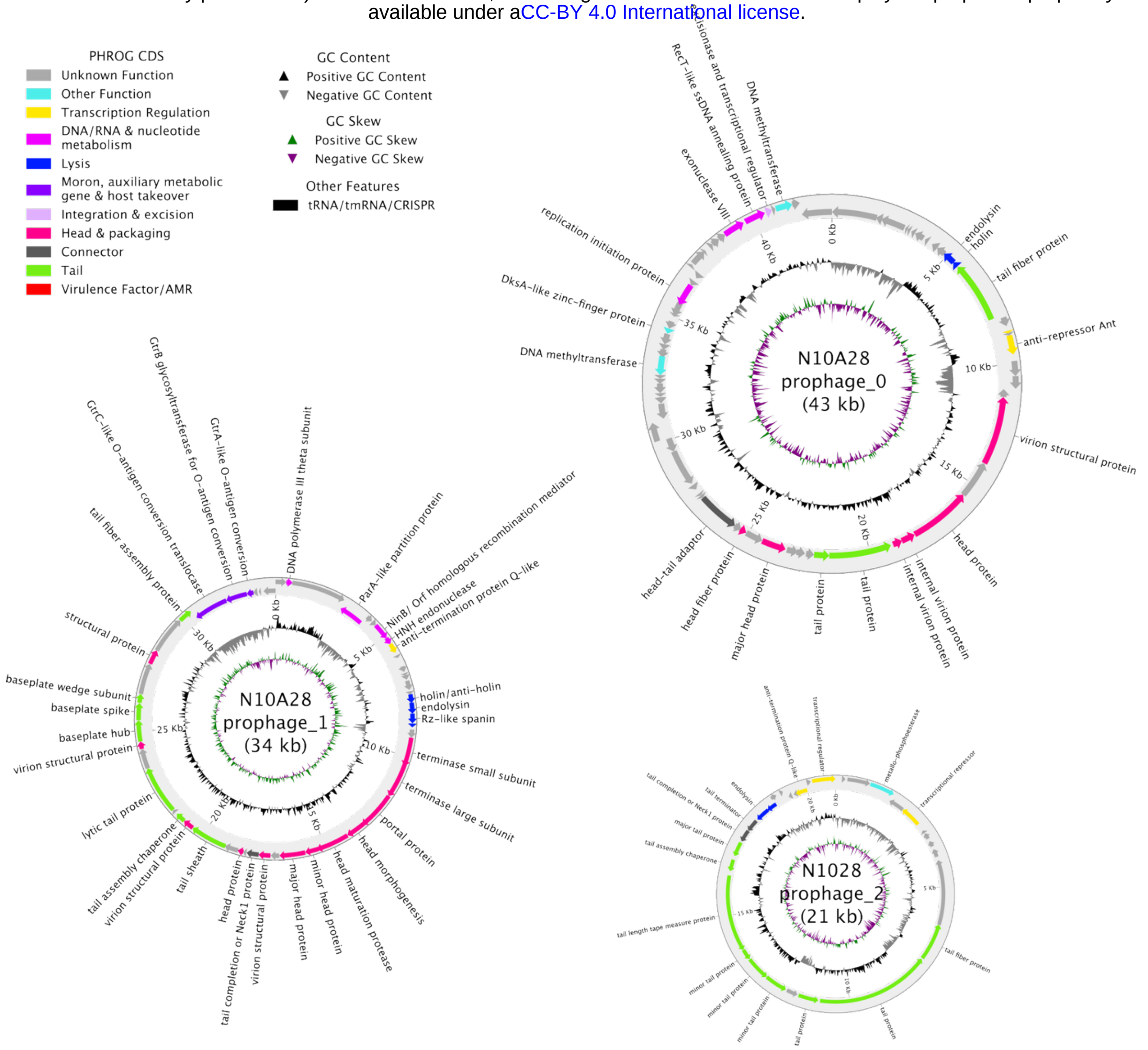

Fig. S8. Three predicted prophage-like structures in the *Serratia marcescens* N10A28 genome (GenBank: CP033623.1). Genomic coordinates: 1,400,308–1,434,310 (Prophage_1); 2,928,641–2,949,521 (Prophage_2); 3,342,031–3,385,057 (Prophage_0).

bioRxiv preprint doi: https://doi.org/10.64898/2025.12.27.695564; this version posted December 27, 2025. The copyright holder for this preprint (which was not certified by peer review) is the author/funder, who has granted bioRxiv a license to display the preprint in perpetuity. It is made available under aCC-BY 4.0 International license.

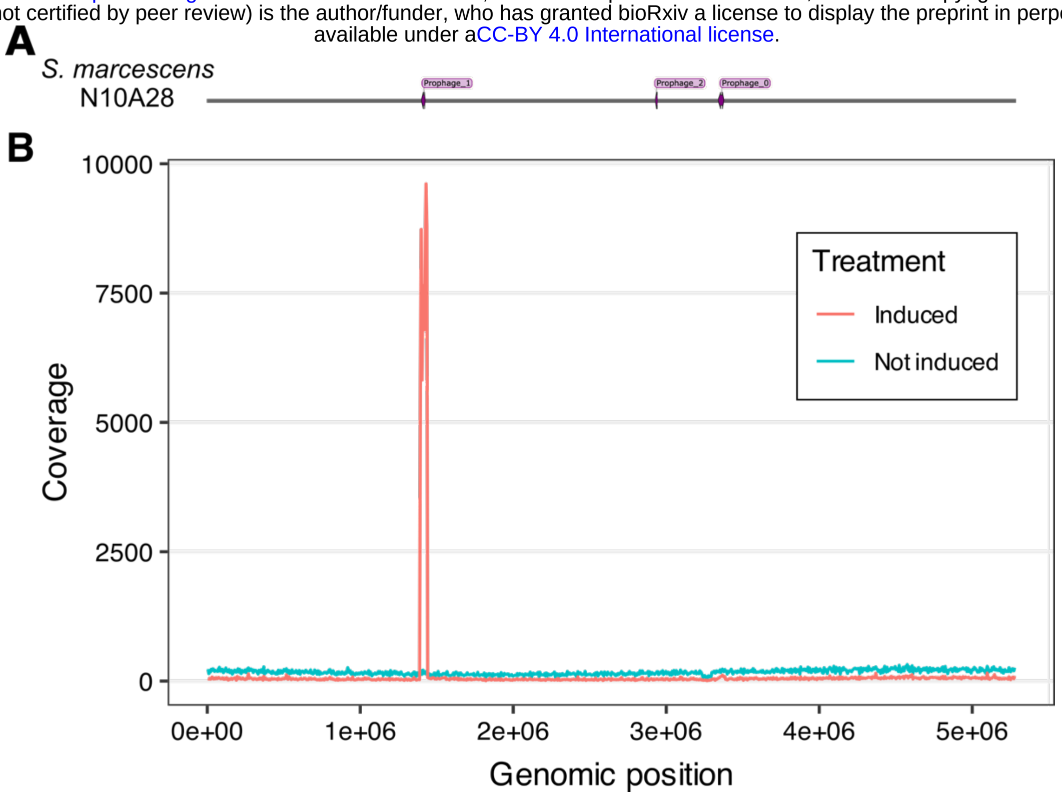

Fig. S9. MMC induced a prophage region in *S. marcescens* N10A28. (A) Purple loci showing positions of the three prophage-like structures in the genome. (B) MMC (0.5 µg/mL) treatment for 5 hours induced lytic replication of Prophage_1; the induced genomic region (1,390,922–1,435,747) encompasses the predicted region (1,400,308–1,434,310). The other two prophage-like regions were not induced. Without MMC, all prophage-like structures were not induced.

bioRxiv preprint doi: https://doi.org/10.64898/2025.12.27.695564; this version posted December 27, 2025. The copyright holder for this preprint (which was not certified by peer review) is the author/funder, who has granted bioRxiv a license to display the preprint in perpetuity. It is made available under aCC-BY 4.0 International license.

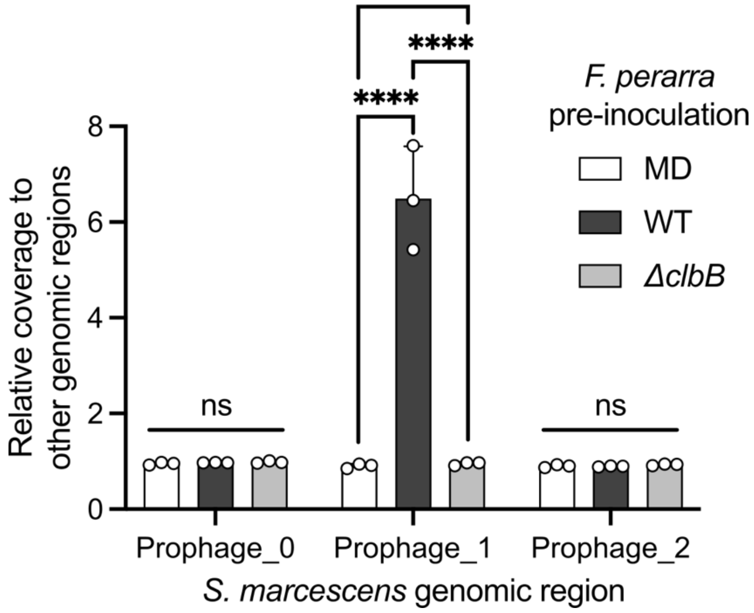

Fig. S10. Relative coverage of *S. marcescens* prophage-like regions to other genomic regions in bee guts pre-colonized with *F. perrara* WT, *ΔclbB* or MD. Prophage_1 was induced by *F. perrara* WT but not by *F. perrara ΔclbB*. The other two prophage-like structures were not induced under all tested conditions. Statistical differences were assessed using a one-way ANOVA with a Tukey's multiple comparisons test (Graphpad Prism). Statistical significance: ****, $p < 0.0001$; ns, not significant.

bioRxiv preprint doi: https://doi.org/10.64898/2025.12.27.695564; this version posted December 27, 2025. The copyright holder for this preprint (which was not certified by peer review) is the author/funder, who has granted bioRxiv a license to display the preprint in perpetuity. It is made available under aCC-BY 4.0 International license.

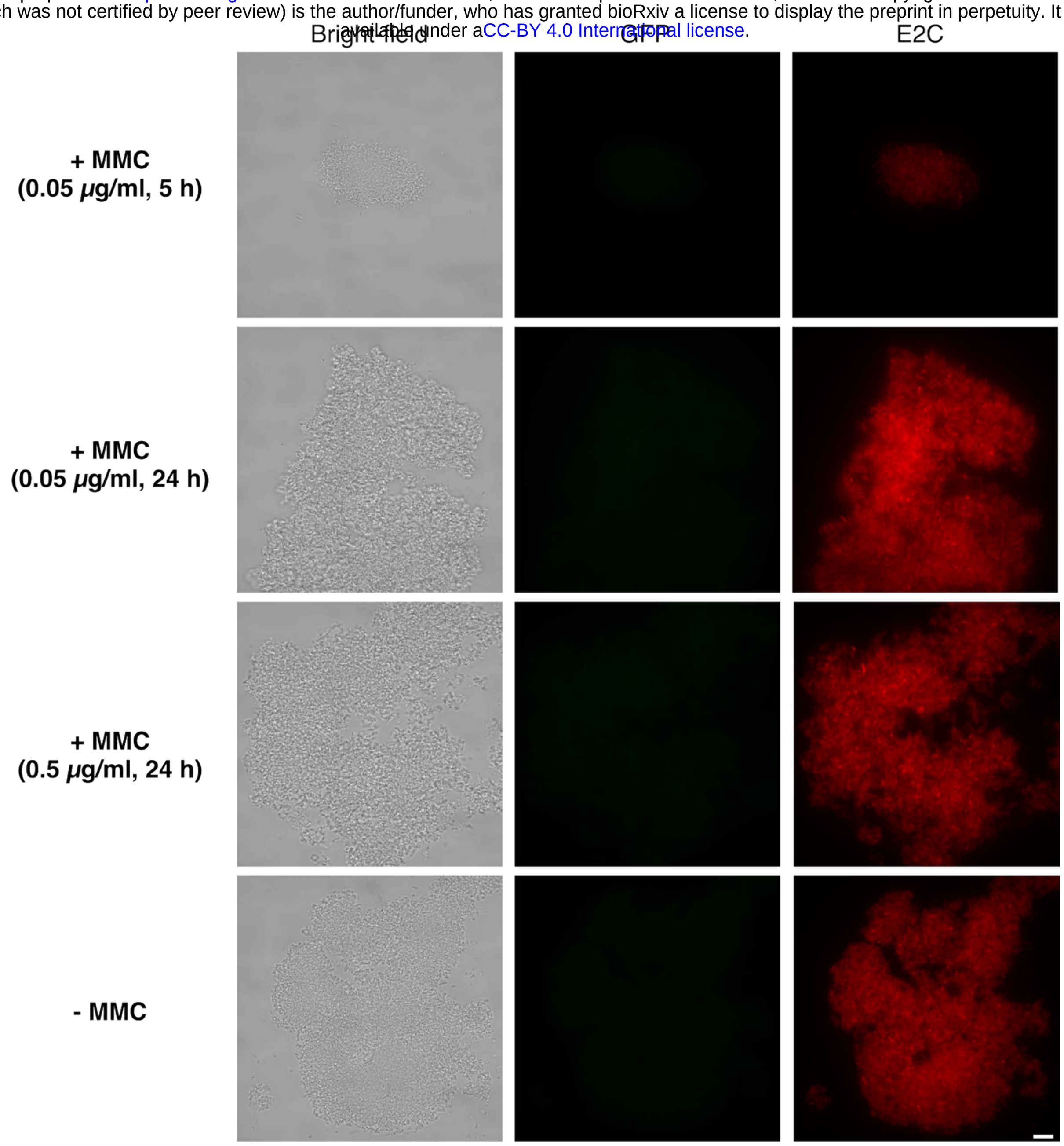

Fig. S11. MMC treatment failed to induce GFP signal in *S. alvi* wkB2 carrying pSL1-E2C-B2P$_{recA}$eGFP. Scale bar = 10 μm.

bioRxiv preprint doi: https://doi.org/10.64898/2025.12.27.695564; this version posted December 27, 2025. The copyright holder for this preprint (which was not certified by peer review) is the author/funder, who has granted bioRxiv a license to display the preprint in perpetuity. It is made available under aCC-BY 4.0 International license.

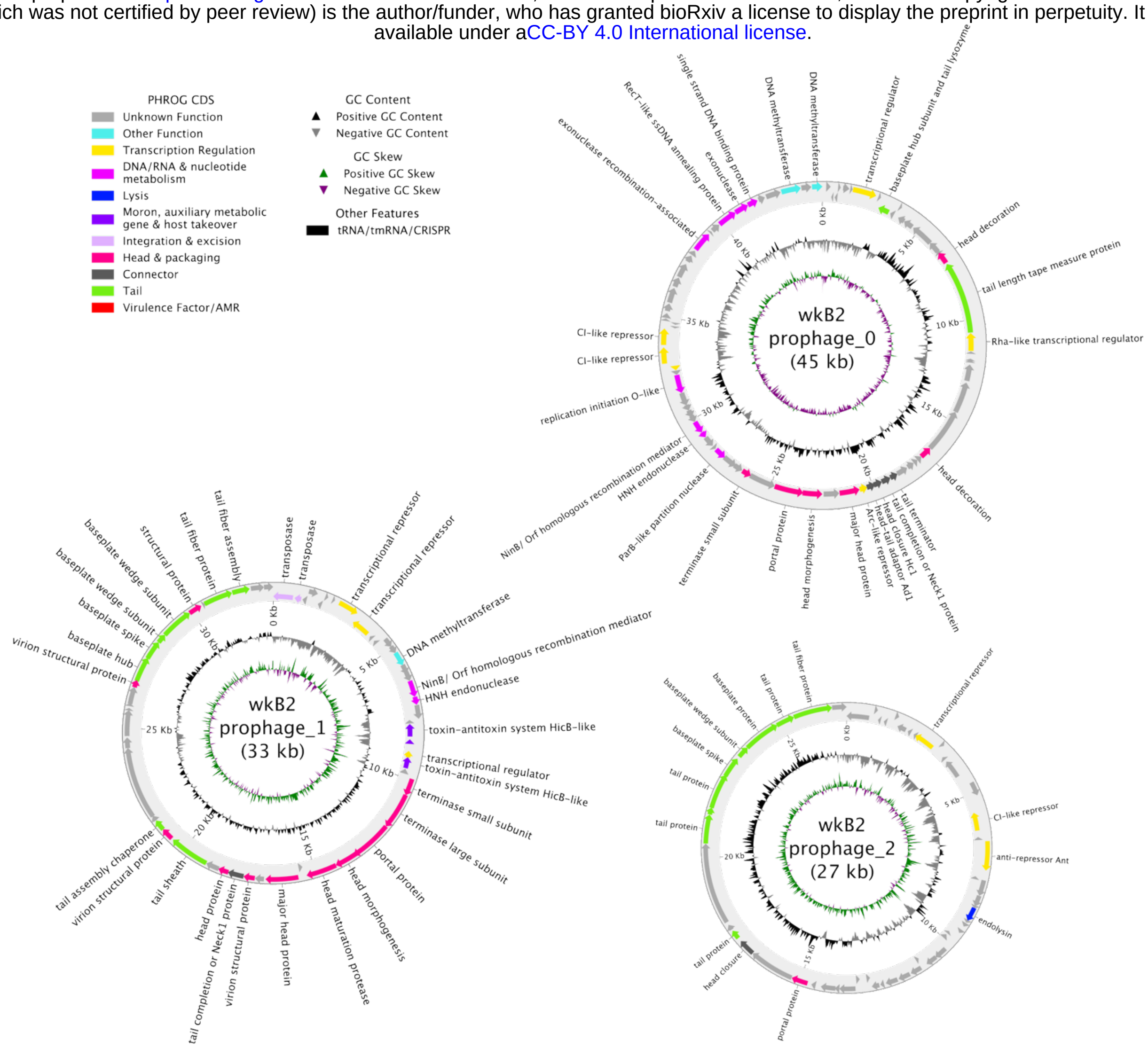

1042

1043 **Fig. S12.** Predicted prophage-like structures in the *Snodgrassella alvi* wkB2 genome
1044 (CP007446.1). Genomic coordinates: 547,445–580,557 (Prophage_1); 1,033,694–1,060,775
1045 (Prophage_2); 2,046,103–2,091,026 (Prophage_0).

bioRxiv preprint doi: https://doi.org/10.64898/2025.12.27.695564; this version posted December 27, 2025. The copyright holder for this preprint (which was not certified by peer review) is the author/funder, who has granted bioRxiv a license to display the preprint in perpetuity. It is made available under aCC-BY 4.0 International license.

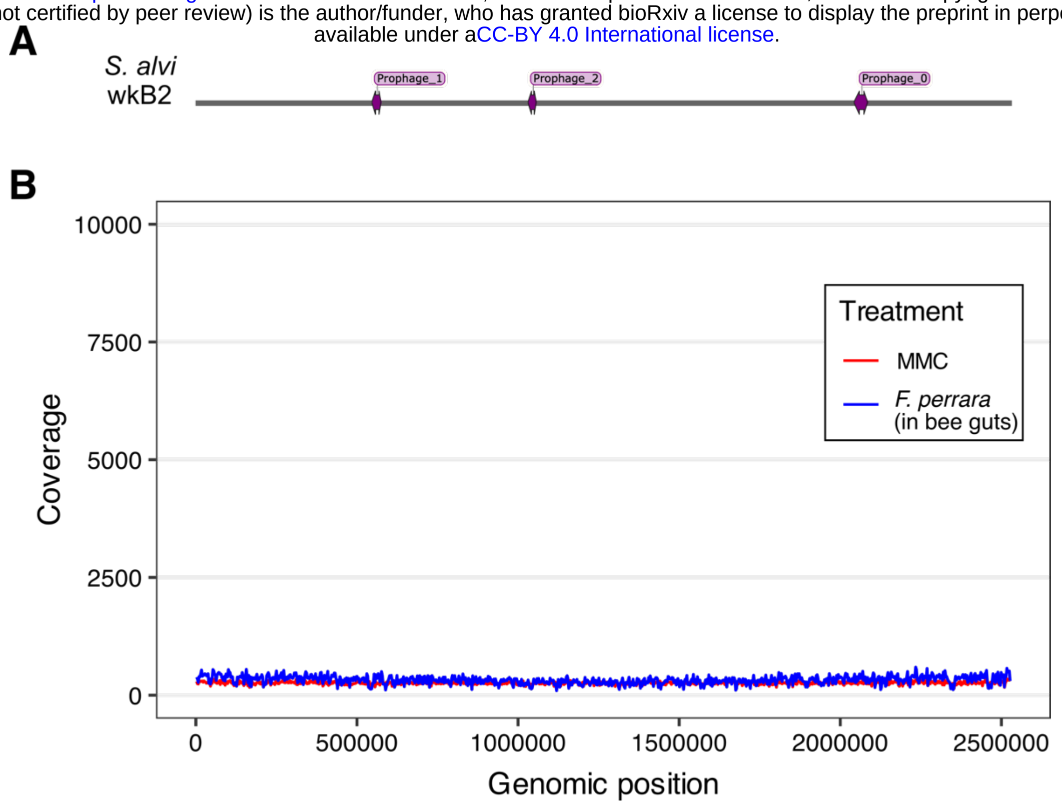

Fig. S13. MMC and *F. perrara* failed to induce lytic replication of prophage-like regions in *S. alvi* wkB2. (A) Purple loci show positions of the three prophage-like structures in the genome. (B) MMC (0.5 µg/mL) treatment for 5 hours or *F. perrara* pre-inoculation did not show prophage induction in wkB2. Bees were pre-inoculated with *F. perrara* WT, after 6 days, inoculated with *S. alvi ΔclbS*, and hindguts were collected after another 4 days.

bioRxiv preprint doi: https://doi.org/10.64898/2025.12.27.695564; this version posted December 27, 2025. The copyright holder for this preprint (which was not certified by peer review) is the author/funder, who has granted bioRxiv a license to display the preprint in perpetuity. It is made available under aCC-BY 4.0 International license.

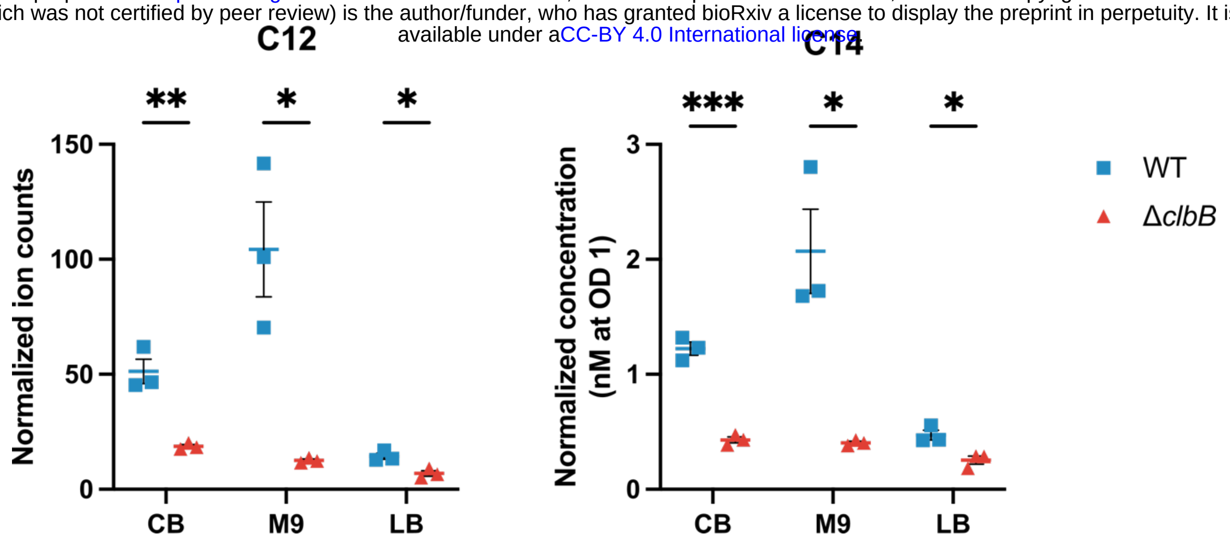

Fig. S14. LC–MS measurement of *N*-lauryl-D-Asn (C12 prodrug) and *N*-myristoyl-D-Asn (C14 prodrug) in cell pellets of *F. perrara* WT and *ΔclbB* cultured with different media. Data are mean ± SEM; $n = 3$ biological replicates. Statistical differences between WT and *ΔclbB* were assessed using a two-tailed unpaired t-test (Graphpad Prism). Statistical significance: ***, $p < 0.001$; **, $p < 0.01$; *, $p < 0.05$. For the M9 groups, strains were cultured in CB first and cell pellets were recollected, washed, and resuspended in M9 medium. *F. perrara* showed very weak growth in LB medium (final OD ~1/10 of that in CB).

bioRxiv preprint doi: https://doi.org/10.64898/2025.12.27.695564; this version posted December 27, 2025. The copyright holder for this preprint (which was not certified by peer review) is the author/funder, who has granted bioRxiv a license to display the preprint in perpetuity. It is made available under aCC-BY 4.0 International license.

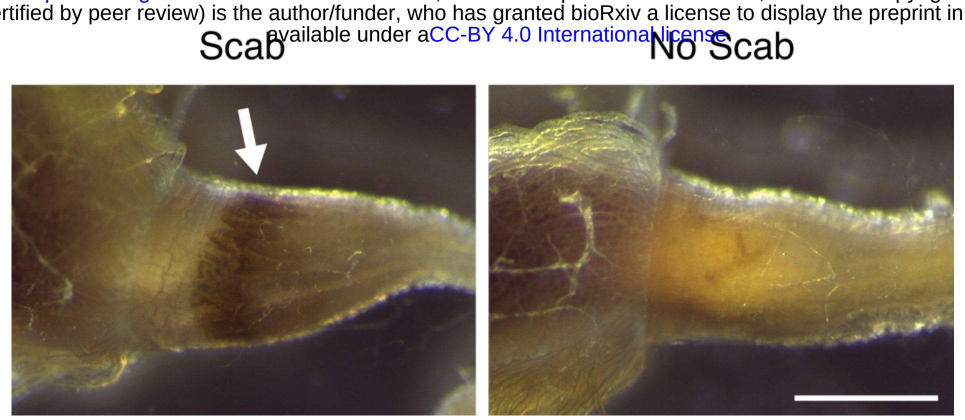

Fig. S15. Scab-like phenotype in the pylorus of the bee gut caused by *F. perrara* colonization. Arrow indicates the melanized region. Scale bar = 0.5 mm.

bioRxiv preprint doi: https://doi.org/10.64898/2025.12.27.695564; this version posted December 27, 2025. The copyright holder for this preprint (which was not certified by peer review) is the author/funder, who has granted bioRxiv a license to display the preprint in perpetuity. It is made available under aCC-BY 4.0 International license.

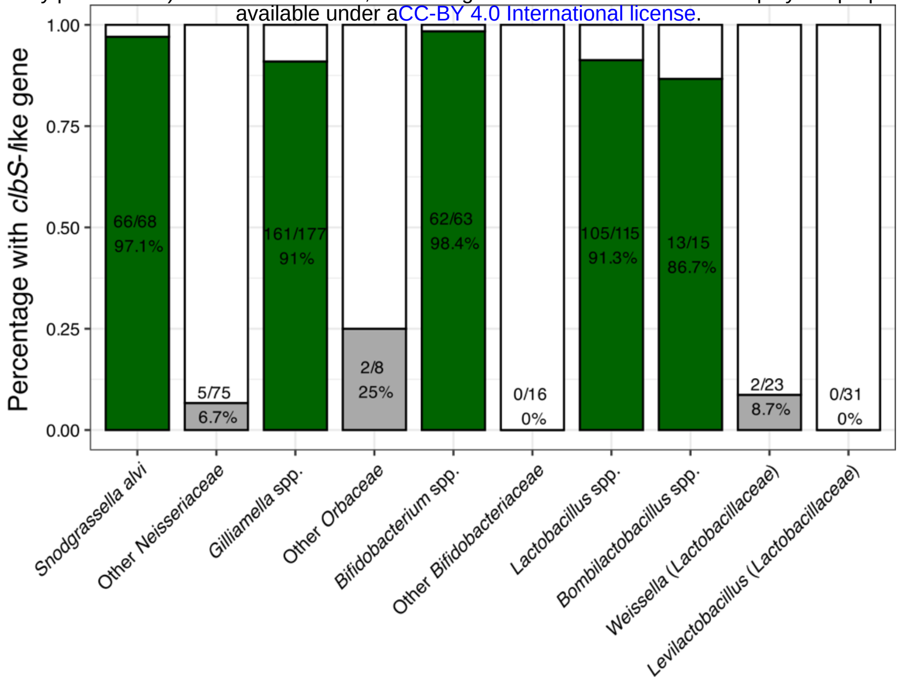

Fig. S16. Presence of *clbS*-like gene in genomes of bee gut core taxa (in green) and closely related taxa from other environments (in grey). Positive/total genome number and percent are given in the plot. Genomes and sequences are detailed in Supplementary Data S1.

bioRxiv preprint doi: https://doi.org/10.64898/2025.12.27.695564; this version posted December 27, 2025. The copyright holder for this preprint (which was not certified by peer review) is the author/funder, who has granted bioRxiv a license to display the preprint in perpetuity. It is made available under aCC-BY 4.0 International license.

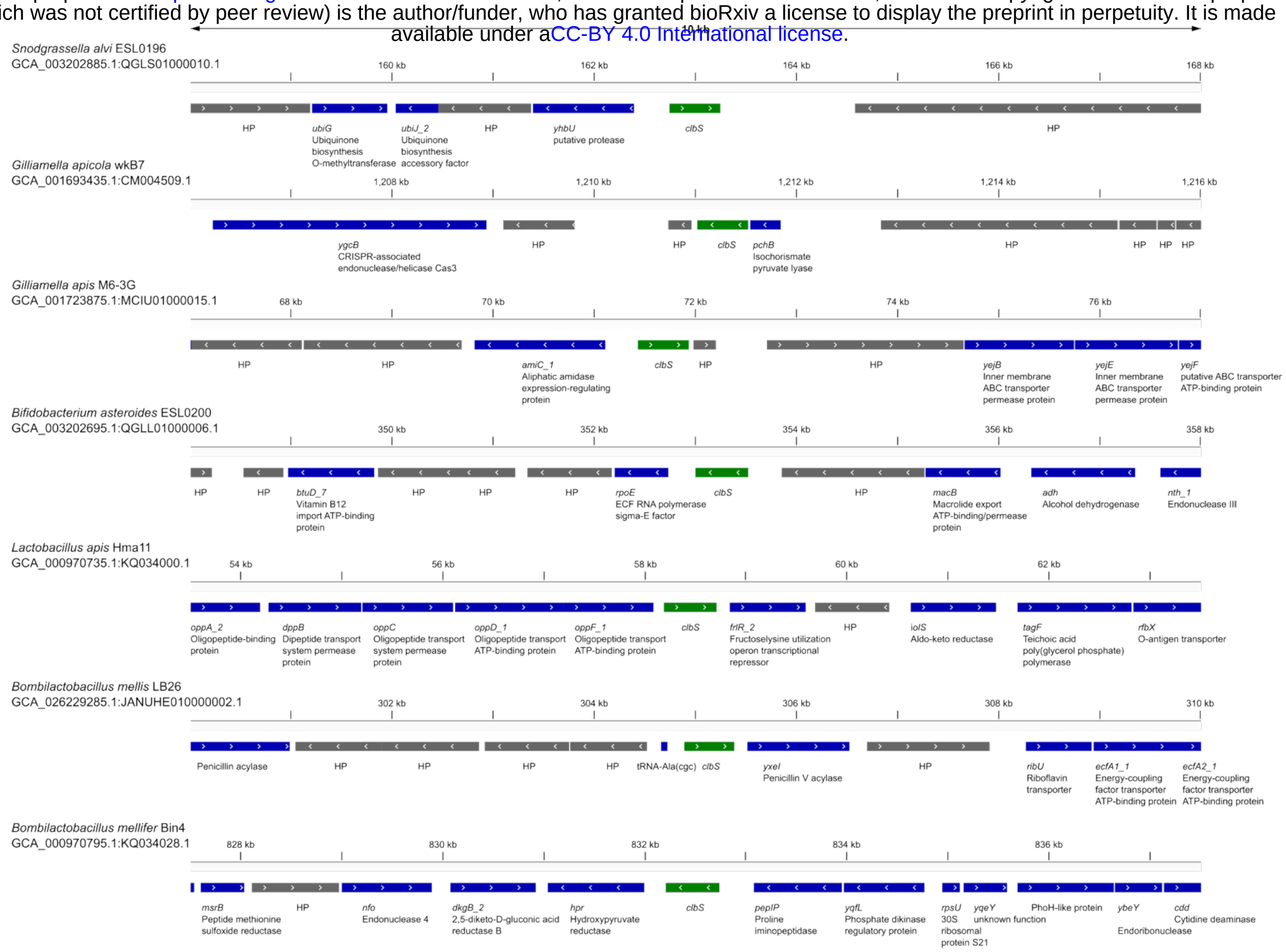

Fig. S17. Gene maps of the tested *clbS* homologs in Fig. 3B. The *clbS* genes are in green. Neighboring genes with annotated functions are in blue (gene abbreviations and names are given), and those encoding hypothetical proteins (HPs) are in grey.

bioRxiv preprint doi: https://doi.org/10.64898/2025.12.27.695564; this version posted December 27, 2025. The copyright holder for this preprint (which was not certified by peer review) is the author/funder, who has granted bioRxiv a license to display the preprint in perpetuity. It is made available under aCC-BY 4.0 International license.

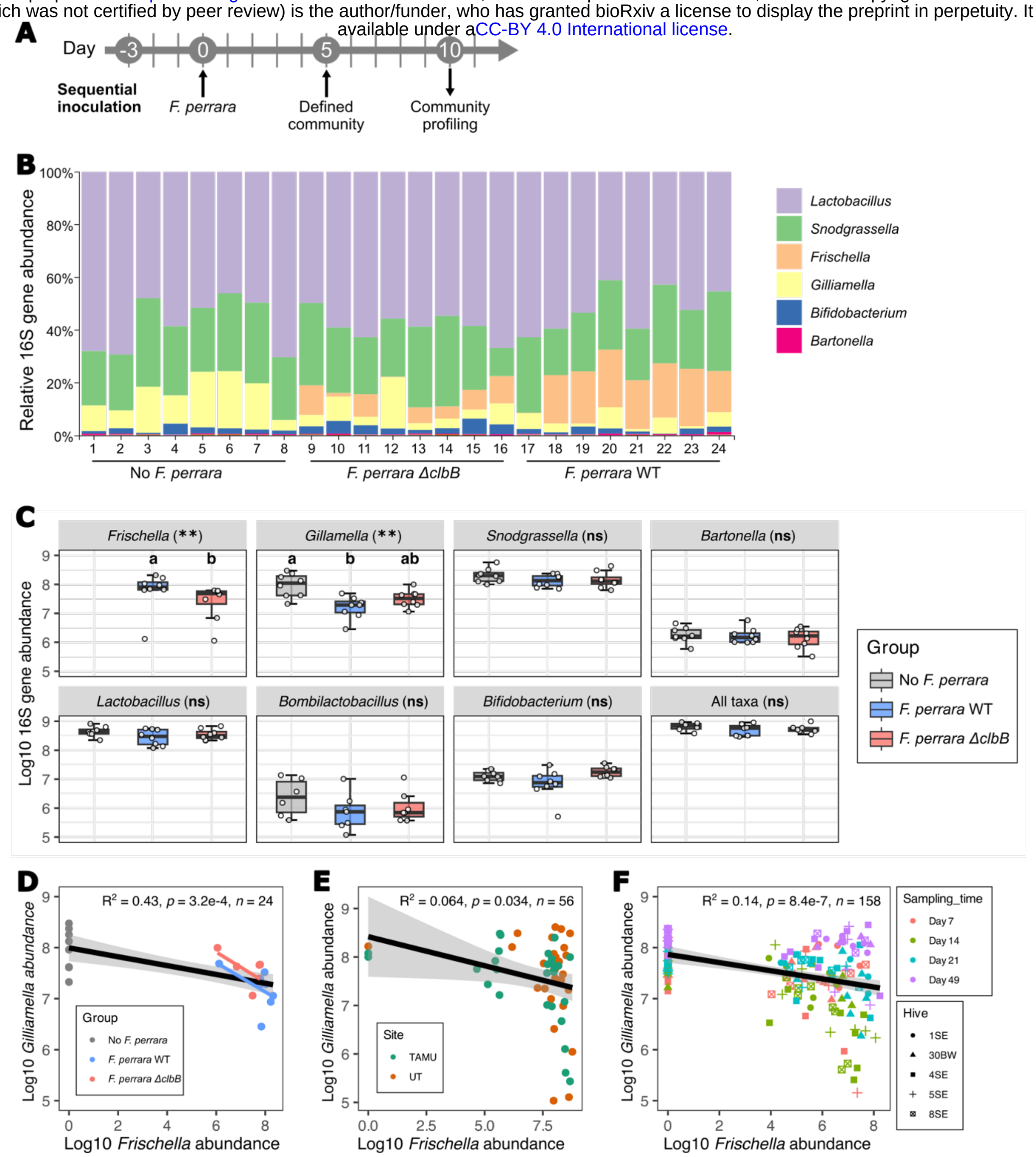

Fig. S18. Levels of *Frischella* and *Gilliamella* are negatively correlated in honeybee guts, but *Frischella* presence does not otherwise affect community composition. (A) Timeline of the sequential inoculation of *F. perrara* and a defined community of honeybee gut bacterial symbionts. (B) Relative abundance of different taxa (classified into genus-level) in the defined community assay. (C) Absolute abundance of different taxa in the defined community assay. Wilcoxon rank sum test indicated a statistically significant difference between *F. perrara* WT and *ΔclbB* (p = 0.007). Kruskal–Wallis test showed no statistical significance in other taxa except *Gilliamella* (χ^2 = 9.695, df =2, p = 0.008, n = 24). Post-hoc pairwise comparisons between *Gilliamella* groups were conducted using Dunn's test. Statistical significance: **, $p < 0.01$; ns, not significant. Groups with different letters are significantly different ($\alpha = 0.05$). (D) *F. perrara* and *Gilliamella* spp. showed negative correlation in the defined community assay. (E) and (F) Negative correlation between *Frischella* and *Gilliamella* in the gut of hive sourced bees. Data from Powell *et al.*, 2021 and 2023 (*9, 10*).

Supplementary Tables

Table S1. Bacterial strains used in this study.

Species	Strain	Antibiotic Marker	Source
Frischella perrara	PEB0191	-	(*11*)
Frischella perrara	PEB0191 *ΔclbB*	-	(*12*)
Snodgrassella alvi	wkB2[a]	-	(*13*)
Snodgrassella alvi	wkB2 *ΔclbS*	*ampR*	This study
Serratia marcescens	N10A28	-	(*10*)
Gilliamella apicola	wkB7[a]	-	(*13*)
Gilliamella apis	M6-3G[a]	-	(*14*)
Bifidobacterium asteroides	wkB338[a]	-	(*15*)
Lactobacillus helsingborgensis	wkB8[a]	-	(*16*)
Lactobacillus kullabergensis	wkB10[a]	-	(*16*)
Bombilactobacillus mellis	Hon2N[a]	-	(*17, 18*)
Bombilactobacillus mellifer	Bin4N[a]	-	(*17, 18*)
Bartonella apis	PEB0150[a]	-	(*19*)
Escherichia coli	DH5α	-	NEB #C2987H
Escherichia coli	MFDpir	-	(*20*)
Escherichia coli	BW25113	-	(*21*)

[a] Strains included in the defined community.

Table S2. Plasmids used in this study.

Plasmid	Function	Antibiotic Marker	Source
pSL1-GFP	Constitutive expression of GFP under CP25 promoter	Kan	(*22*)
pSL1-RCP	Constitutive expression of Red Chrome Protein under CP25 promoter	Kan	(*22*)
pSL1-E2C	Constitutive expression of E2-Crimson under CP25 promoter	Kan	(*22*)
pSL1-E2C-N10P_{recA}eGFP	Constitutive expression of E2-Crimson under CP25 promoter and inducible expression of eGFP by DNA damage under *recA* promoter of *Serratia marcescens* N10A28	Kan	This study
pSL1-E2C-B2P_{recA}eGFP	Constitutive expression of E2-Crimson under CP25 promoter and inducible expression of eGFP by DNA damage under *recA* promoter of *Snodgrassella alvi* wkB2	Kan	This study
pBeloBAC11	Empty bacterial artificial chromosome (BAC)	Cm	NEB
BAC-*pks*	BAC with *pks* genomic island for colibactin production	Cm	(*23*)
P_R-*lux*	Bioluminescent reporter for DNA damage	Kan	(*24*)
pTrcHis2_A	Empty pTrc vector for *clbS*-like genes cloning	Amp	Thermo Fisher Scientific
pTrc-*clbS*	Expression of WT *clbS* from *E. coli* CFT073 under Trc promoter	Amp	This study
pTrc-*clbS*$_{Snod}$	Expression of *clbS*-like gene from *Snodgrassella alvi* ESL0196	Amp	This study
pTrc-*clbS*$_{Gillia1}$	Expression of *clbS*-like gene from *Gilliamella apicola* wkB7	Amp	This study
pTrc-*clbS*$_{Gillia2}$	Expression of *clbS*-like gene from *Gilliamella apis* M6-3G	Amp	This study
pTrc-*clbS*$_{Bifido}$	Expression of *clbS*-like gene from *Bifidobacterium asteroids* ESL0200	Amp	This study
pTrc-*clbS*$_{Lacto}$	Expression of *clbS*-like gene from *Lactobacillus apis* Hma11	Amp	This study
pTrc-*clbS*$_{Bombi1}$	Expression of *clbS*-like gene from *Bombilactobacillus mellis* LB26	Amp	This study
pTrc-*clbS*$_{Bombi2}$	Expression of *clbS*-like gene from *Bombilactobacillus mellifer* Bin4	Amp	This study

Table S3. Primers used in this study.

Primer	Sequence (5' to 3')	Function
***clbS* gene deletion in wkB2**[a]		
B2_clbS-L-f	CCAGAGCCTGCCGTAATA	Amplify left homology arm and full cassette
B2_clbS-L-r	CCGAAAAGTGCCACCTGTTGGTTCTCCCTACCTTCAG	Amplify left homology arm
B2_clbS-R-f	GGTGAAGATCCTTTTTGATAATCATTAATTATTAGCAATAATCTGGTC	Amplify right homology arm
B2_clbS-R-r	GCTTGGCAGAAACTGATAGA	Amplify right homology arm and full cassette
ampR-f	CAGGTGGCACTTTTCGG	Amplify ampicillin resistant cassette
ampR-r	GATTATCAAAAAGGATCTTCACC	Amplify ampicillin resistant cassette
B2_clbS-S-f[b]	CCGTAGTTTTATTTTCCG	Verify *clbS* deletion mutant
B2_clbS-S-r[b]	CGTGACCAGATTATTGCT	Verify *clbS* deletion mutant
**pSL1-E2C-N10P_{recA}eGFP and -B2P_{recA}eGFP plasmid construction**[c]		
psl1-f	GGCTACCGTCTCACCAGCTTTGGCAGTTTATTCTTGACATG	Amplify pSL1-E2C backbone
psl1-r	GGCTACCGTCTCATCTCAAGGGGCCTGTCAGA	Amplify pSL1-E2C backbone
N10-PrecA-f	GGCTACGGTCTCAGAGAATTAGGCTTGATACTGTATGACCATACAG	Amplify N10P_{recA}eGFP
B2-PrecA-f	GGCTACGGTCTCAGAGACGTAAATTAGCATTTGAATGAAGTCATTACAG	Amplify B2P_{recA}eGFP
ampR-psl1-r	GGCTACGGTCTCACTGGGATTATCAAAAAGGATCTTCACCTAGATCC	Amplify N10P_{recA}eGFP and B2P_{recA}eGFP
**Community profiling**		
Hyb515F_rRNA[d]	TCGTCGGCAGCGTCAGATGTGTATAAGAGACAGGTGYCAGCMGCCGCGGTA	Amplify 16S rRNA gene V4 region
Hyb806R_rRNA[d]	GTCTCGTGGGCTCGGAGATGTGTATAAGAGACAGGGACTACHVGGGTWTCTAAT	Amplify 16S rRNA gene V4 region
Hyb_Fnn_i5[e]	AATGATACGGCGACCACCGAGATCTACAC NNNNNNNN TCGTCGGCAGCGTC	Barcode 16S amplicons
Hyb_Rnn_i7[e]	CAAGCAGAAGACGGCATACGAGAT NNNNNNNN GTCTCGTGGGCTCGG	Barcode 16S amplicons
27F[f]	AGAGTTTGATCCTGGCTCAG	Quantify 16S gene copy numbers
355R[f]	CTGCTGCCTCCCGTAGGAGT	Quantify 16S gene copy numbers

[a] Sequences in green are overlapping regions in the fusion PCR reaction.[b] The screen primers amplify ~650 bp fragment from genomic DNA template of WT and ~1.2 kbp fragment from that of *clbS* gene knockout mutant. [c] Sequences in blue are enzyme recognition sites of BsaI or BsmBI. [d] Primer sequences from Wang and Qian, 2009 (*25*). [e] Illumina Nextera sequences, "NNNNNNNN" signifies 8 bp unique sample barcodes. [f] Primer sequences from Castillo *et al.*, 2006 (*26*).

Table S4. Synthesized DNA fragments.

Gene block	Sequence (5’ to 3’)
**Cassettes for DNA damage reporting**[a]	
N10P$_{recA}$eGFP	ATTAGGCTTGATACTGTATGACCATACAGTATAATTAGTGACATTTCCTGCACAACATACATTCAGTGGCAGTGTGGGGTAACAC<br>CGGCATTGCGAAACGAAGGAGCAAAAATGGTGAGCAAGGGCGAGGAGCTGTTCACCGGGGTGGTGCCCATCCTGGTCGAGCTG<br>GACGGCGACGTAAACGGCCACAAGTTCAGCGTGTCCGGCGAGGGCGAGGGCGATGCCACCTACGGCAAGCTGACCCTGAAGTT<br>CATCTGCACCACCGGCAAGCTGCCCGTGCCCTGGCCCACCCTCGTGACCACCCTGACCTACGGCGTGCAGTGCTTCAGCCGCTAC<br>CCCGACCACATGAAGCAGCACGACTTCTTCAAGTCCGCCATGCCCGAAGGCTACGTCCAGGAGCGCACCATCTTCTTCAAGGAC<br>GACGGCAACTACAAGACCCGCGCCGAGGTGAAGTTCGAGGGCGACACCCTGGTGAACCGCATCGAGCTGAAGGGCATCGACTT<br>CAAGGAGGACGGCAACATCCTGGGGCACAAGCTGGAGTACAACTACAACAGCCACAACGTCTATATCATGGCCGACAAGCAGA<br>AGAACGGCATCAAGGTGAACTTCAAGATCCGCCACAACATCGAGGACGGCAGCGTGCAGCTCGCCGACCACTACCAGCAGAAC<br>ACCCCCATCGGCGACGGCCCCGTGCTGCTGCCCGACAACCACTACCTGAGCACCCAGTCCGCCCTGAGCAAAGACCCCAACGAG<br>AAGCGCGATCACATGGTCCTGCTGGAGTTCGTGACCGCCGCCGGGATCACTCTCGGCATGGACGAGCTGTACAAGTAACTGTCA<br>GACCAAGTTTACTCATATATACTTTAGATTGATTTAAAACTTCATTTTTAATTTAAAAGGATCTAGGTGAAGATCCTTTTTGATAA<br>TC
B2P$_{recA}$eGFP	CGTAAATTAGCATTTGAATGAAGTCATTACAGCTTTGTATAACCTTTTGCAGACGGGCATTCAGCTACTAAGTAACAGATAACAA<br>AATCGTATAATTCAAATAGATAGCATAAATAAGCTTTTGCTAAACATCTAATCATAGGCTTATGAGATAATTAACTTTATTAAGT<br>TAATCATACAGTACAAGCGAGCATATCAATATGGTGAGCAAGGGCGAGGAGCTGTTCACCGGGGTGGTGCCCATCCTGGTCGAG<br>CTGGACGGCGACGTAAACGGCCACAAGTTCAGCGTGTCCGGCGAGGGCGAGGGCGATGCCACCTACGGCAAGCTGACCCTGAA<br>GTTCATCTGCACCACCGGCAAGCTGCCCGTGCCCTGGCCCACCCTCGTGACCACCCTGACCTACGGCGTGCAGTGCTTCAGCCGC<br>TACCCCGACCACATGAAGCAGCACGACTTCTTCAAGTCCGCCATGCCCGAAGGCTACGTCCAGGAGCGCACCATCTTCTTCAAGG<br>ACGACGGCAACTACAAGACCCGCGCCGAGGTGAAGTTCGAGGGCGACACCCTGGTGAACCGCATCGAGCTGAAGGGCATCGAC<br>TTCAAGGAGGACGGCAACATCCTGGGGCACAAGCTGGAGTACAACTACAACAGCCACAACGTCTATATCATGGCCGACAAGCA<br>GAAGAACGGCATCAAGGTGAACTTCAAGATCCGCCACAACATCGAGGACGGCAGCGTGCAGCTCGCCGACCACTACCAGCAGA<br>ACACCCCCATCGGCGACGGCCCCGTGCTGCTGCCCGACAACCACTACCTGAGCACCCAGTCCGCCCTGAGCAAAGACCCCAACG<br>AGAAGCGCGATCACATGGTCCTGCTGGAGTTCGTGACCGCCGCCGGGATCACTCTCGGCATGGACGAGCTGTACAAGTAACTGT<br>CAGACCAAGTTTACTCATATATACTTTAGATTGATTTAAAACTTCATTTTTAATTTAAAAGGATCTAGGTGAAGATCCTTTTTGAT<br>AATC
**Representatives of *clbS*-like genes from honebee gut symbionts**[b]	
clbS<br>(*E. coli* CFT073)	ATGGCCGTACCATCCTCGAAGGAAGAACTTATCAAGGCGATCAATTCTAACTTCTCGCTGTTGAACAAGAAGTTGGAGAGCATT<br>ACACCACAGCTGGCGTTCGAACCACTTCTTGAGGGCCATGCGAAAGGAACAACCATCTCAGTCGCCAACTTGGTTAGCTATCTTA<br>TCGGCTGGGGGGAATTGGTTCTTCACTGGCACGATCAAGAAGCTAAAGGCAAAACTATTATCTTTCCCGAAGAAGGGTTTAAGT<br>GGAATGAGCTGGGACGCTTGGCTCAAAAGTTCTACCGTGATTACGAAGACATTACAGAATACGAGGTTCTGTTAGCTCGCCTTAA<br>AGAGAATAAACAGCAATTAGTGGCGTTGATCGAACGTTTCTCAAACGATGAGCTTTATGGTAAGCCCTGGTATAACAAATGGAC

	CCGTGGACGTATGATCCAATTTAATACTGCATCCCCGTACAAAAATGCAAGTGGTCGTTTGAACAAGTTGCAGAAGTGCTTAGCT<br>GAATAA
clbS$_{Snod}$<br>(*S. alvi* ESL0196)	ATGGCAATCCCCGAGAGTAAGCAAGAGCTCATCGAAGCAATAAACAAGAATTACACTCTGTTGACGAAGAAACTCGCGGCGGTT<br>CCAGAGCAAAAGGCATACTTACCATTAATGGAAGGGCACGCGAAGGGGACGATGATGTCGGCCGCCCAATTAGTCAGCTACCTG<br>ATCGGTTGGGGTGAGTTAGTTCTTTCGTGGCACAAGCAAGAGCAATCGGGCCAAAAGATAGCGTTCCCAGAGGCTGGTTACAAG<br>TGGAACCAGTTGGGCCTTCTTGCGCAAAAGTTCTACAGAGACTACCAAGACATTACTGATTTTAAGCAACTGTTGAGCCTGTTAG<br>AAACTAATAAGAAGAACCTCATATCCCTGATAGACAGTTTCAGCAACGAGGAATTATACGGTTCGCCGTGGCACGAGAAGTATA<br>CTCGTGGTCGTATGATCCAATTCAACACGTCGTCACCGTACAAGAACGCAACGGGGCGCCTCAACAAGCTGCTGAAGCAGATAG<br>CTGAGTGA
clbS$_{Gillia1}$<br>(*G. apicola* wkB7)	ATGGCCTACGCTTCCAAGCAGCAACTCCTGGACGAGATCAATAAGACGGCGAAATTATTCATCAACGAGTTCACAGACCTGCTG<br>GAGAGCGAGAAGGACCTCTTAATCGATGGCGTGGACCGCACACCCGCGCAGATGATAGCATACCAACTGGGGTGGCTGAAGCT<br>GGTAAAGTCCTGGGACGACGATGAGCTGGCTGGGAAGACCCCGGAACTGCCGGCTCCGGAATACAAGTGGAACCGACTTGGGA<br>GCCTTTACCAGACGTTTTACGACACATACCAGGGCTTATCACTTAGCGAGTTGATACAGAGCTTCAAGCAGCAGATAACCATATG<br>GAATAACTGGATAGAAAGCCTGTCAGACGACACACTCTTCATAAAGGACGTGCGTAACTGGACAAAGCCGTACCCGGAGTCCTG<br>GACGGTTGCGCGGTTCATACATATTAACTCCGTCGCTCCGTTCAAGTCTTTCCGTAGCAAGATTCGCAAATGGAAGAAGCTGAAT<br>AACCCTGTCTGA
clbS $_{Gillia2}$<br>(*G. apis* M6-3G)	ATGAACTATGCAAACAAGCAGCAGCTTTTGGACGAGATCAACAAGACCGCGAAACTTTTCATAGACGAGTTTTCGGGGCTGACG<br>GAAGCTGAGAAGGACAAGCTGATCGAGGGAGTCGACCGGACACCGGCGCAAATGATCGCATACCAGCTGGGCTGGCTGGACTT<br>AGTCAAGTCCTGGGACGACGCCGAGCTGGCAGGTCAGACTCCAATCCTGCCGGCCGAGGGCTATAAGTGGAACCAACTGGGAG<br>AGCTCTATCAGCACTTCTACAAGATCTACCAGGACGACTCCCTTCAGGAGTTGATTCAGCTTTTCAAGCAAAAGCTGGCCACATG<br>GAACAGTTGGATCGAATCCCTTGACGACAACACCCTGTTCACGAAGGACGCAAGAAACTGGACCAAACCGTACCCATCTGCTTG<br>GCCGGTAGCCCGGTTCATACATATCAACAGCGTAGCTCCGTTCAAGAGCTTCCGTACGAAGATTCGCAAGTGGAAGAAGTTAAA<br>CTCTCACTGA
clbS$_{Bifido}$<br>(*B. asteroids* ESL0200)	ATGGCCCGACCAACGACAAAAGAGGAGCTGATTAAAGCAGCAGACGACGGGTTTGATAAGCTGGTTGCCCTTCTGGAACCATTA<br>ACTCCAGAGGAGCGTAACGGTCGTTTCTCTTTTGACCTCTCCAAAGAAAAGGGTGCGCACTGGGCTAGAGATCGGAATATTAAA<br>GACGTCTTGGTCCACTTATACGAATGGCACCAACTCTTACTGAATTGGGTTGAAGATAATCAACAAGGTCGGGCACATGATTTCC<br>TGCCGGATGGGTATAATTGGCGGAACTATGGTGAGATGAACGTAAAATTAAGAGATGAACACGACGATACGACGTATGACCAA<br>GCTCTTAATTTATTCATTGAGTCGCACAAGAAGGTTATGGCACTCGCCCAGTCTTTTACGAATGAGCAATTGTTTACGAAAGGTA<br>CACTGCCGTGGACCGGTAATTCGACTCTGGGTAGCGCATTTATTAGTAGCACATCATCCCACTATGATTGGGCTTTAAAGAAAAT<br>TCGCAAATATCTGAAAGGCCGGAAGTGA
clbS$_{Lacto}$<br>(*L. apis* Hma11)	ATGGCGCGTCCGACCAACAAGACAGACCTTATCGAGGCTAGCAACACGGCTTACCAGAAAATCGTGGACCTTATAGCGAGTCTC<br>CCGGAAGAAGCGCGCACCGCGGATTTCGACTTCGACACCTCGAAATTGAAAGAGGCGCACTGGAAGCGTGATCATAATGTGCGC<br>GATGTCCTTGCACACCTGTACGAGTGGCAGAAGCTGCTCATCGATTGGATCGACAGCAACCAGGCGGGGAAGCCGAAGGAGTAC<br>CTGCCGGAAGGTTACAACTGGAGAAACTACGGTGAGATGAATCAGGAATTCTGGAAGAATGATCAGAGCATACCGTTAGAGGA<br>GATGGAAGAACTTCTGGCGCAGTCTCACAAGAAGACTATGGCACTCATAGATTCGTACACTAACGACGAGCTGTTTCAGAAGAA

	CGTGTTTCCGTGGACCGGGAACAACGCACTCGGCACGTACTTCATAGCTAACACCAGTTCGCATTACGAGTGGGCGCTGAAGAA<br>ACTTCGCAAGTACAAACGTTCAATTAAATGA
clbS$_{Bombi1}$<br>(*B. mellis* LB26)	ATGCGAACTTATCAGAACAAACAAGAGCTGATACAGGCGATCCAGACTCAATACCAAAAGTACATCACGGAGTTCGCAAACATC<br>CCTGAGGCACAGAAGGACCTTAGCGTCGAGGGCGTTGATAAGACCCCGTCACAGAACTTAGCATATCAGCTGGGTTGGTTAAAT<br>TTAATGCTGGAATGGGACCAGAAGGAGAAGCAGGGCCTGGCCGTTCAGACTCCCACGCCAGACTACAAATGGAACCAACTTGGT<br>GGTCTGTACCAGAGCTTCTACAATAAGTACGGCAACACGACCCTGCAGAAACAGGTTGACGAGCTGAACGCCCTTGTGGATCAG<br>TTTACGGACTGGATCGACAGTCTGTCCGAAGCGGAGTTGTTCGAGGTTGGCCAACGTAAGTGGGCAACCACCAAAGCGAGATGG<br>CCCTTGTGGCGTTGGATCGACATAAACACAGTTAGTCCATTTACCACCTTCCGTACCAAGATTAGAAAATGGAAGAAGCTGGCAT<br>TGTGA
clbS$_{Bombi2}$<br>(*B. mellifer* Bin4)	ATGGCACGCCCGACCACCAAAACCGAACTGATTAACACCAGTCAGACCAACTATCAGAAAATTAAAGATCTGATTGCGGCGCTG<br>CCGCCGGCGGCGCGCACCGCCGATTTCAACTTCGATATTAGCAGCCTGAAAGAAGCCCATTGGCAGCGCGATCACAACGTGCGT<br>GATGTGCTGATTCATCTGTACGAATGGCAGAAACTGCTGCTGACCTGGATTCAGAACAACCAGGCGGGCCAGAAACAGGATTTT<br>CTGCCGGCGGGCTACAACTGGCGCACCTACGGCCAGATGAACCTGGCGTTTTGGCAGAAACATCAGACCACCAGCCTGGAAACC<br>GCCGAAAAACTGCTGGATCAGACCCACCAGCAGGTGATGAGCATTCTGGATACCTTTAGCGAAGATCAGCTGTTTCAGAAACAC<br>ATTTATCCGTGGACCGGCAACAATACGCTGGGCAGCTATTTTATTGCCAATACCACCTCGCACTATGATTGGGCGCTGAAAAAAC<br>TGCGTAAATATCAGCGCCGCCTGCCGCGTACGAACTAA

[a] DNA sequence colors correspond to promoter (orange, from N10A28 or wkB2), eGFP (green), and terminator (purple, from pBTK501).

[b] Codon-optimized sequences for *E. coli* expression.

bioRxiv preprint doi: https://doi.org/10.64898/2025.12.27.695564; this version posted December 27, 2025. The copyright holder for this preprint (which was not certified by peer review) is the author/funder, who has granted bioRxiv a license to display the preprint in perpetuity. It is made available under aCC-BY 4.0 International license.

Supplementary Data

Data S1. Presence of *clbS*-like genes in genomes of bee gut bacterial taxa and closely related taxa from other environments.

Supplementary References

1. P. Engel, M. I. Vizcaino, J. M. Crawford, Gut symbionts from distinct hosts exhibit genotoxic activity via divergent colibactin biosynthesis pathways. *Appl Environ Microbiol* **81**, 1502-1512 (2015).
2. K. M. Ellegaard, P. Engel, New reference genome sequences for 17 bacterial strains of the honey bee gut microbiota. *Microbiol Resour Announc* **7**, e00834-18 (2018).
3. L. Guo, J. Tang, M. Tang, S. Luo, X. Zhou, Reactive oxygen species are regulated by immune deficiency and Toll pathways in determining the host specificity of honeybee gut bacteria. *Proc Natl Acad Sci U S A* **120**, e2219634120 (2023).
4. M. I. Steele, E. V. S. Motta, T. Gattu, D. Martinez, N. A. Moran, The gut microbiota protects bees from invasion by a bacterial pathogen. *Microbiol Spectr* **9**, e0039421 (2021).
5. R. D. Horak, S. P. Leonard, N. A. Moran, Symbionts shape host innate immunity in honeybees. *Proc Biol Sci* **287**, 20201184 (2020).
6. J. C. Arthur *et al.*, Intestinal inflammation targets cancer-inducing activity of the microbiota. *Science* **338**, 120-123 (2012).
7. C. Harnack *et al.*, Short-term mucosal disruption enables colibactin-producing *E. coli* to cause long-term perturbation of colonic homeostasis. *Gut Microbes* **15**, 2233689 (2023).
8. M. Olier *et al.*, Genotoxicity of *Escherichia coli* Nissle 1917 strain cannot be dissociated from its probiotic activity. *Gut Microbes* **3**, 501-509 (2012).
9. J. E. Powell *et al.*, The microbiome and gene expression of honey bee workers are affected by a diet containing pollen substitutes. *PLoS One* **18**, e0286070 (2023).
10. J. E. Powell, Z. Carver, S. P. Leonard, N. A. Moran, Field-realistic tylosin exposure impacts honey bee microbiota and pathogen susceptibility, which is ameliorated by native gut probiotics. *Microbiol Spectr*, e0010321 (2021).
11. P. Engel, W. K. Kwong, N. A. Moran, *Frischella perrara* gen. nov., sp. nov., a gammaproteobacterium isolated from the gut of the honeybee, *Apis mellifera*. *Int J Syst Evol Microbiol* **63**, 3646-3651 (2013).
12. K. Schmidt *et al.*, Integration host factor regulates colonization factors in the bee gut symbiont *Frischella perrara*. *Elife* **12**, e76182 (2023).
13. W. K. Kwong, N. A. Moran, Cultivation and characterization of the gut symbionts of honey bees and bumble bees: description of *Snodgrassella alvi* gen. nov., sp. nov., a member of the family *Neisseriaceae* of the *Betaproteobacteria*, and *Gilliamella apicola* gen. nov., sp. nov., a member of *Orbaceae* fam. nov., *Orbales* ord. nov., a sister taxon to the order *'Enterobacteriales'* of the *Gammaproteobacteria*. *Int J Syst Evol Microbiol* **63**, 2008-2018 (2013).
14. H. Zheng *et al.*, Metabolism of toxic sugars by strains of the bee gut symbiont *Gilliamella apicola*. *mBio* **7**, e01326-16 (2016).
15. H. Zheng *et al.*, Division of labor in honey bee gut microbiota for plant polysaccharide digestion. *Proc Natl Acad Sci U S A* **116**, 25909-25916 (2019).

bioRxiv preprint doi: https://doi.org/10.64898/2025.12.27.695564; this version posted December 27, 2025. The copyright holder for this preprint (which was not certified by peer review) is the author/funder, who has granted bioRxiv a license to display the preprint in perpetuity. It is made available under aCC-BY 4.0 International license.

16. W. K. Kwong, A. L. Mancenido, N. A. Moran, Genome sequences of *Lactobacillus* sp. strains wkB8 and wkB10, members of the Firm-5 clade, from honey bee guts. *Genome Announc* **2**, e01176-14 (2014).
17. J. Zheng *et al.*, A taxonomic note on the genus *Lactobacillus*: Description of 23 novel genera, emended description of the genus *Lactobacillus* Beijerinck 1901, and union of *Lactobacillaceae* and *Leuconostocaceae*. *Int J Syst Evol Microbiol* **70**, 2782-2858 (2020).
18. T. C. Olofsson, M. Alsterfjord, B. Nilson, E. Butler, A. Vasquez, Lactobacillus apinorum sp. nov., *Lactobacillus mellifer* sp. nov., *Lactobacillus mellis* sp. nov., *Lactobacillus melliventris* sp. nov., *Lactobacillus kimbladii* sp. nov., *Lactobacillus helsingborgensis* sp. nov. and *Lactobacillus kullabergensis* sp. nov., isolated from the honey stomach of the honeybee *Apis mellifera*. *Int J Syst Evol Microbiol* **64**, 3109-3119 (2014).
19. F. H. Segers, L. Kesnerova, M. Kosoy, P. Engel, Genomic changes associated with the evolutionary transition of an insect gut symbiont into a blood-borne pathogen. *ISME J* **11**, 1232-1244 (2017).
20. L. Ferrieres *et al.*, Silent mischief: bacteriophage Mu insertions contaminate products of *Escherichia coli* random mutagenesis performed using suicidal transposon delivery plasmids mobilized by broad-host-range RP4 conjugative machinery. *J Bacteriol* **192**, 6418-6427 (2010).
21. T. Baba *et al.*, Construction of *Escherichia coli* K-12 in-frame, single-gene knockout mutants: the Keio collection. *Mol Syst Biol* **2**, 2006 0008 (2006).
22. Y. Li, S. P. Leonard, J. E. Powell, N. A. Moran, Species divergence in gut-restricted bacteria of social bees. *Proc Natl Acad Sci U S A* **119**, e2115013119 (2022).
23. J. P. Nougayrede *et al.*, *Escherichia coli* induces DNA double-strand breaks in eukaryotic cells. *Science* **313**, 848-851 (2006).
24. J. E. Silpe, J. W. H. Wong, S. V. Owen, M. Baym, E. P. Balskus, The bacterial toxin colibactin triggers prophage induction. *Nature* **603**, 315-320 (2022).
25. Y. Wang, P. Y. Qian, Conservative fragments in bacterial 16S rRNA genes and primer design for 16S ribosomal DNA amplicons in metagenomic studies. *PLoS One* **4**, e7401 (2009).
26. M. Castillo *et al.*, Quantification of total bacteria, enterobacteria and lactobacilli populations in pig digesta by real-time PCR. *Vet Microbiol* **114**, 165-170 (2006).
